# Photosensitizing Drugs and Risk of Skin Cancer in Women—A Prospective Population‐Based Study

**DOI:** 10.1111/phpp.70013

**Published:** 2025-03-18

**Authors:** Gustav Boelsgaard Christensen, Johan Kappelin, Jenny Sandgren, Kari Nielsen, Åsa Ingvar

**Affiliations:** ^1^ Department of Dermatology Skåne University Hospital Lund Sweden; ^2^ Lund University Skin Cancer Research Group, Dermatology, Department of Clinical Sciences, Lund Lund University Lund Sweden; ^3^ Department of Dermatology Landskrona Hospital Landskrona Sweden; ^4^ Clinical Studies Sweden—Forum South Skåne University Hospital Lund Sweden; ^5^ Department of Dermatology Helsingborg Hospital Helsingborg Sweden

**Keywords:** basal cell carcinoma, melanoma, photosensitizing drugs, prospective, squamous cell carcinoma

## Abstract

**Background:**

Several widely used drugs have photosensitizing properties, and much research has been conducted to find associations between their use and the risk of developing cutaneous malignant melanoma (cM), basal cell carcinoma (BCC) or cutaneous squamous cell carcinoma (cSCC), often with conflicting results.

**Objective:**

To assess whether the use of commonly prescribed photosensitizing drugs increases skin cancer risk.

**Methods:**

Analyses were performed using a large cohort of women, with prospectively collected information on phenotypic traits and sun exposure. Comprehensive information on pharmaceutical treatments and skin cancer occurrence was obtained through national registries. Drugs with photosensitizing properties were grouped according to the Anatomical Therapeutic Chemical system in nine groups, and associations between the use of such drugs were investigated using multivariable Cox regression analysis. The number of retrieved daily doses was analyzed to test the dose–response relationship.

**Results:**

Hormone replacement therapy significantly increased the risk of BCC (hazard ratio [HR] 1.24; 95% confidence interval [CI]: 1.11–1.39), cSCC (HR 1.23; 95% CI: 1.03–1.47) and cM (HR 1.31; 95% CI: 1.01–1.69), with estrogen driving this risk. There was a trend of increased risk of BCC and cM with higher doses of estrogen treatment. Subgroup analysis among those using diuretics showed that loop diuretics were associated with increased cSCC risk (HR 1.6; 95% CI: 1.3–2.0), including a positive association between risk and dose. Furthermore, increased risks of BCC (HR 1.25; 95% CI: 1.09–1.44) and cM (HR 1.41; 95% CI: 1.03–1.93) were associated with thiazide use. NSAIDs showed a possible curvilinear association to BCC and cSCC.

**Conclusions:**

Estrogen treatment increased the risk of all investigated skin cancers. Among those using diuretics, loop diuretics increased the risk of cSCC, and thiazide use increased the risk of BCC. We suggest that physicians should advise female patients prescribed estrogen, thiazides, or loop diuretics to limit their sun exposure.

## 1 Introduction

The main risk factor for skin cancer is ultraviolet radiation (UVR) including both solar radiation and radiation from tanning devices [1, 2, 3, 4]. The incidences of cutaneous malignant melanoma (cM), cutaneous squamous cell carcinoma (cSCC) and basal cell carcinoma (BCC) are steadily increasing in fair‐skinned populations [4, 5, 6]. Phenotypic and environmental factors interact and determine the risk of developing any cancer, including skin cancer. Fair‐skinned people, such as those from Northern Europe, are more sensitive to UVR and have a greater risk of developing skin cancer [7, 8]. Theoretically, factors that enhance UVR effects can increase skin cancer risk. One such possible factor is the use of drugs with photosensitizing properties [9, 10, 11].

The pharmacodynamics of these drugs include various chemical changes in the skin that cause individuals to become more sensitive to UVR through phototoxic reactions [9, 10, 11, 12]. Several of these drugs are used to treat common chronic diseases such as hypertension and diabetes.

Studies of the relationships between exposure to photosensitizing drugs and skin cancer have yielded conflicting results. The most studied drugs are antihypertensives, including diuretics [13, 14, 15, 16, 17, 18, 19, 20, 21, 22, 23]. Notably, the FDA put forward a label change for the diuretic hydrochlorothiazide (HCTZ) due to a slight increase in the risk of non‐melanoma skin cancer [24]. The limitations of these studies include a retrospective design, a lack of adjustments for confounders, and the inability to find causative associations [14, 16, 17, 25, 26].

To assess whether the use of established prescribed photosensitizing drugs indeed increases the risk of certain skin cancers, we used the MISS cohort of 29,000 Swedish women and prospectively collected data describing known risk factors for analysis.

## 2 Materials and Methods

### 2.1 Setting

We used the prospective Melanoma Inquiry of Southern Sweden (MISS) study cohort, established in 1990–1992, which is described in detail elsewhere [2, 27, 28, 29, 30]. 40,000 Swedish‐born women (1000 per calendar year from 1925 to 1965) were chosen through random computerized selection from the National Population Registry and were invited to join the initial cohort. Non‐responders were sent one reminder. At baseline, all women lived in Southern Sweden's healthcare region and had no history of invasive cancer. Participants provided written informed consent.

At baseline, participants answered a validated detailed questionnaire [30, 31, 32, 33] asking about common risk factors for skin cancer such as constitutional characteristics, sun exposure habits, socioeconomic factors, educational level, and smoking habits. Exposure to UVR was assessed by asking questions about sunburns, sunbathing, sunbed use, outdoor work, and sunny holidays. Follow‐up questionnaires were sent to participants every tenth year. For this study, we extracted data from the baseline and first follow‐up questionnaires.

Using the unique 12‐digit personal registration number assigned to all Swedish citizens, the cohort was linked to the Swedish Cancer Registry, the regional pathology registers, and the Swedish Prescribed Drug Register (SPDR). Participants were followed from 1 January 2008 until either the diagnosis of skin cancer, emigration, death, or the end of follow‐up on 31 December 2018. Women diagnosed with skin cancer before the study began were excluded. Only primary diagnoses of cSCC, BCC, or cM were used in the analyses. Participants were considered to be exposed to the included drugs if one or more prescriptions were registered in their names.

### 2.2 Ethics

The Institutional Ethics Review Board of Lund University and the Swedish Ethical Review Authority approved the study (LU‐34‐1992, LU‐632‐2003, LU‐781‐2005, LU‐849‐2005, LU‐190‐2007, LU‐644‐2015, and LU‐02914‐2022).

### 2.3 Photosensitizing Drugs

Using literature reviews to determine if photosensitizing effects were suspected, we chose to include the following drug classes: antidiabetics, diuretics, β‐blockers, calcium channel blockers (CCB), angiotensin converting enzyme inhibitors (ACEi), angiotensin receptor blockers (ARB), proton pump inhibitors (PPi), NSAIDs, antibiotics, and hormonal replacement therapies (Table 1). The drugs were grouped according to the Anatomical Therapeutic Chemical (ATC) classification system.

**TABLE 1:** Number of exposed, and proportion of diagnosed skin cancer among exposed, categorized by group of medication and skin cancer type.

Photosensitizing medication	ATC classification	Exposed, *n*	BCC, *n* (%)	Exposed, *n*	cSCC, *n* (%)	Exposed, *n*	cM, *n* (%)
Estrogen, progesterone, and combination	G03C, G03D, G03F	8844	526 (5.9)	8926	218 (2.5)	8956	110 (1.2)
Estrogen	G03C	7531	467 (6.2)	7621	200 (2.7)	7652	96 (1.3)
Progesterone	G03D	1083	38 (3.5)	1089	9 (0.8)	1087	5 (0.5)
Estrogen/Progesterone comb.	G03F	1565	81 (5.2)	1574	24 (1.5)	1575	18 (1.1)
Diuretics	C03A, C03BA, C03C, C03D, C09BA, C03EA	7121	413 (5.8)	7214	239 (3.4)	7239	76 (1.0)
Thiazides	C03AA, C03AB, C03AH, C03EA	3848	267 (6.9)	3883	125 (3.2)	3900	52 (1.3)
Loop diuretics	C03CA, C03EB, C03CB	3817	157 (4.1)	3889	127 (3.3)	3914	27 (0.7)
Other diuretics	C03BA, C03D, C09BA	1971	93 (4.7)	2012	59 (3.0)	2026	15 (0.7)
CCB	C01B, C08C, C09BB	5213	266 (5.1)	5300	145 (2.8)	5326	51 (1.0)
ACEi, ARB	C09A, C09B, C09C	7232	354 (4.9)	7352	181 (2.5)	7384	66 (0.9)
β‐blockers	C07A	7115	388 (5.5)	7201	197 (2.8)	7226	77 (1.1)
Antibiotics	J01M, J01A, J01E, J01XE	11,071	551 (5.0)	11,229	264 (2.4)	11,260	117 (1.0)
Antidiabetics	A10BB, A10BA02	1611	69 (4.3)	1636	39 (2.4)	1634	9 (0.6)
PPi	A02BC	9072	427 (4.7)	9204	217 (2.4)	9232	82 (0.9)
NSAID	M01A	12,389	638 (5.1)	12,512	268 (2.2)	12,533	138 (1.1)
Total		21,062	1308 (6.2)	21,062	528 (2.5)	21,062	257 (1.2)

Abbreviations: β‐blockers, beta‐blockers; ACEi, angiotensin converting enzyme inhibitors; ARB, angiotensin receptor blockers; CCB, calcium channel blockers; NSAID, non‐steroidal anti‐inflammatory drugs; PPi, proton pump inhibitors.

### 2.4 Statistical Methods

Multivariable Cox regression analyses were performed using complete cases, controlling for known risk factors (described in Table S1), and using drug exposures as time‐dependent covariates. Adjusted hazard ratios (HRs) and 95% confidence intervals (CIs) for the risk associated with each of the studied skin cancers with ever‐never use of each photosensitizing drug were analyzed. For drug groups significantly associated with skin cancer risk, or drugs with previous evidence of an association with skin cancer, subgroup analyses were performed, if possible. Finally, the prescription quantity of defined daily dose (PDDD) was extracted from the SPDR to evaluate the dose‐effect relationship. We define PDDD as the number of standard daily doses of a drug, when used for its primary indication, included in a single prescription. The sum of a person's PDDD was divided by the duration of the treatment (last date—first date of prescription +30 days that is a common length of a prescription) and split into quartiles. Each skin cancer was analyzed separately, and each drug group, subgroup, and dose variable were added to a base model with 13 possible confounding variables (phenotypic traits and UVR exposure) that were selected from the questionnaires (Table S1). Tests for proportional hazards were performed for statistically significant variables. Using a two‐sided statistical test, statistical significance was recognized when *p* < 0.05. Bonferroni adjustments were also performed in the ever‐never analyses by dividing the p‐value by the number of models analyzed (*n* = 19), and statistical significance was recognized when the Bonferroni‐adjusted *p* < 0.05. Analyses were performed using SAS Enterprise Guide, version 8.3 (SAS Institute Inc.) [34].

## 3 Results

**FIGURE 1:**
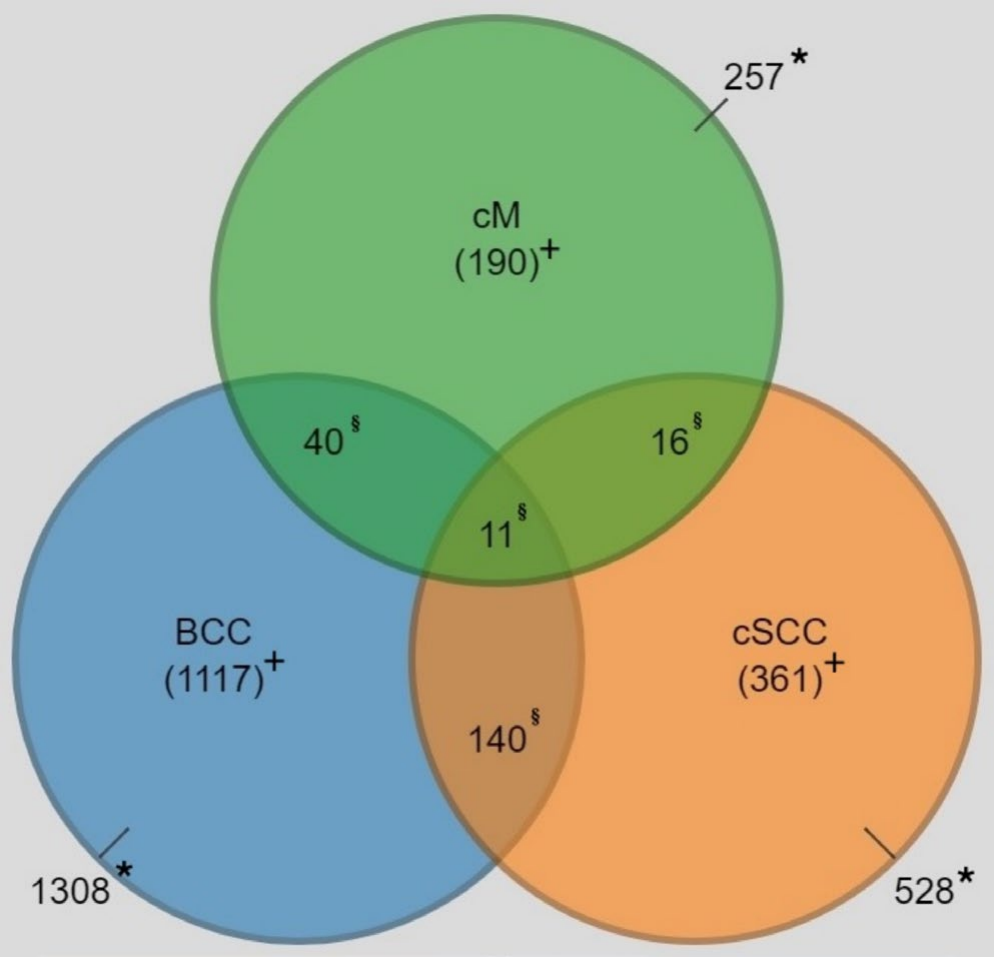
Number of individuals divided by skin cancer type and overlap between skin cancer types, presented as the number of individuals. cM, cutaneous melanoma; BCC, basal cell carcinoma; cSCC, cutaneous squamous cell carcinoma. *Total number of persons diagnosed with a specific skin cancer type. ^+^Number of persons diagnosed exclusively with a specific skin cancer type. ^§^Number of persons diagnosed with two or three different skin cancer types, categorized according to specific combinations.

At baseline, 40,000 women were invited to the MISS study, and a total of 29,515 agreed to participate. We excluded 3405 participants due to emigration, death, or prior skin cancer before the start date of this study, leaving 26,110 women of whom 21,062 had complete data. During the study, 1308 primary BCC, 528 primary SCC, and 257 primary cM were registered. Of the 1875 participants that developed skin cancer, 1668 (89%) developed only one of the three skin cancers. The most common combination was between BCC and SCC, in all 140 participants. Eleven participants had cM, BCC, and SCC, as shown in Figure 1.

### 3.1 Photosensitizing Drug Use

Groups of photosensitizing drugs and the number of exposed individuals in total and by skin cancer type are shown in Table 1. Because drug intake was followed until the diagnosis of a specific skin cancer, the number of participants in the drug group varied somewhat throughout the study period. Because the group with primary diagnoses of cM is the smallest of the three, it will have a slightly higher number of individuals in the drug group.

Table 2 shows skin cancer risk as an adjusted HR with a 95% CI for each photosensitizing drug group, compared with the never‐use group. Female hormone use significantly increased the risk of BCC (HR 1.24; 95% CI: 1.11–1.39), cSCC (HR 1.23; 95% CI: 1.03–1.47) and cM (HR 1.31; 95% CI: 1.01–1.69). Sub‐analyses of progesterone, estrogen, and combination treatment revealed that this effect was dominated by the estrogen component. After Bonferroni adjustment, only the association between estrogen use and BCC remained significant.

**TABLE 2:** Risk of skin cancer development in relation to ever‐use compared to never‐use of photosensitizing medication, categorized by group and, when possible, subgroups of medication and skin cancer type, adjusted for known risk factors for skin cancers (described in Table S1).

Photosensitizing medication	BCC	cSCC	cM
	HR (95% CI)^a^	HR (95% CI)^a^	HR (95% CI)^a^
Estrogen, progesterone, and combination	1.24 (1.11–1.39)**	1.23 (1.03–1.47)*	1.31 (1.01–1.69)*
Estrogen	1.25 (1.11–1.40)**	1.23 (1.03–1.48)*	1.35 (1.03–1.75)*
Progesterone	1.23 (0.88–1.72)	1.01 (0.51–2.00)	0.52 (0.21–1.27)
Estrogen/Progesterone comb.	1.22 (0.97–1.53)	1.05 (0.69–1.60)	1.14 (0.70–1.85)
Diuretics	1.12 (0.99–1.27)	1.53 (1.27–1.84)**	1.20 (0.90–1.60)
Thiazides	1.25 (1.09–1.44)**	1.17 (0.95–1.44)	1.41 (1.03–1.93)*
Loop diuretics	0.92 (0.78–1.10)	1.60 (1.29–1.98)**	0.92 (0.61–1.40)
Other diuretics	1.00 (0.81–1.24)	1.30 (0.98–1.71)	0.93 (0.55–1.58)
CCB	1.05 (0.91–1.21)	1.16 (0.95–1.41)	1.16 (0.84–1.61)
ACEi, ARB	0.94 (0.83–1.07)	1.03 (0.85–1.24)	0.96 (0.72–1.30)
β‐blockers	1.00 (0.88–1.13)	1.10 (0.91–1.32)	1.17 (0.88–1.55)
Antibiotics	1.14 (1.02–1.28)*	1.35 (1.13–1.62)**	1.26 (0.97–1.63)
Antidiabetics	0.88 (0.69–1.12)	1.05 (0.76–1.46)	0.64 (0.33–1.24)
PPi	1.02 (0.91–1.15)	1.22 (1.02–1.46)*	0.98 (0.75–1.29)
NSAID	1.12 (1.00–1.25)	1.08 (0.90–1.28)	1.26 (0.98–1.63)

Abbreviations: β‐blockers, beta‐blockers; ACEi, angiotensin converting enzyme inhibitors; ARB, angiotensin receptor blockers; CCB, calcium channel blockers; NSAID, non‐steroidal anti‐inflammatory drugs; PPi, proton pump inhibitors.

^a^ Hazard ratio (HR) of ever‐use of photosensitizing medication compared to never‐use, presented with 95% confidence intervals (CI) regarding basal cell carcinoma (BCC), cutaneous squamous cell carcinoma (cSCC) and cutaneous melanoma (cM).

* Significant *p* < 0.05.

** Significant *p* < 0.05 after Bonferroni correction for multiple testing.

Diuretic use significantly increased the risk of cSCC, but not of BCC or cM. Subgroup analyses showed that the risk of cSCC was significantly associated with the use of loop diuretics (HR 1.6; 95% CI: 1.3–2.0), but not with the other two groups (thiazide diuretics and other diuretics). However, thiazide use increased the risk of BCC (HR 1.25; 95% CI: 1.09–1.44) and cM (HR 1.41; 95% CI: 1.03–1.93). After Bonferroni adjustment, the association between thiazide treatment and the risk of cM became non‐significant.

The other anti‐hypertensive drugs, ACEi, ARB, β‐blockers, and CCB, were not associated with skin cancer risk. Photosensitizing antibiotics (tetracycline, sulfonamides, fluoroquinolones, and nitrofuran derivates) were related to increased risks of BCC (HR 1.14; 95% CI: 1.02–1.28) and cSCC (HR 1.35; 95% CI: 1.13–1.62), the former being non‐significant after Bonferroni adjustment. The risk of cM increased non‐significantly (HR 1.26; 95% CI: 0.97–1.63). Use of PPIs was only associated with an increased risk of cSCC (HR 1.22; 95% CI: 1.02–1.46), but was non‐significant after Bonferroni adjustment.

### 3.2 Dose‐Dependent Risk

To further assess causality, dose‐dependent responses of selected photosensitizing drugs were evaluated (see Table 3). The dose of estrogen showed a positive correlation to the risk of BCC. Compared to never users of estrogen, the risk of BCC increased 60% in the group using the highest dose (HR 1.6; 95% CI: 1.30–1.96). A similar trend was found for the risk of cM but not for cSCC.

**TABLE 3:** Risk of skin cancer development, depending on quartiles of daily dose (number of dispensed daily standard doses divided by duration of treatment), and depicted in relation to never use, categorized by medication group and skin cancer type, adjusted for known risk factors for skin cancers (described in Table S1).

Photosensitizing medication^a^	BCC	cSCC	cM
	HR (95% CI)^b^	HR (95% CI)^b^	HR (95% CI)^b^
Estrogen, progesterone, and combination: 1q	0.96 (0.79–1.16)	1.02 (0.76–1.37)	0.80 (0.49–1.30)
Estrogen, progesterone, and combination: 2q	1.39 (1.17–1.65)^‡^	1.39 (1.07–1.81)***	1.39 (0.94–2.06)
Estrogen, progesterone, and combination: 3q	1.10 (0.91–1.34)	1.14 (0.85–1.54)	1.57 (1.07–2.29)***
Estrogen, progesterone, and combination: 4q	1.68 (1.39–2.03)^‡^	1.46 (1.06–2.01)***	1.58 (1.03–2.41)^‡^
Estrogen: 1q	0.97 (0.79–1.19)	0.99 (0.72–1.37)	0.90 (0.55–1.47)
Estrogen: 2q	1.29 (1.07–1.55)***	1.43 (1.09–1.88)***	1.46 (0.96–2.21)
Estrogen: 3q	1.26 (1.04–1.52)***	1.27 (0.96–1.69)	1.39 (0.92–2.11)
Estrogen: 4q	1.60 (1.30–1.96)^‡^	1.21 (0.85–1.73)	1.76 (1.14–2.72)***
Estrogen/Progesterone combination: 1q	0.86 (0.54–1.36)	0.81 (0.36–1.82)	0.64 (0.21–2.01)
Estrogen/Progesterone combination: 2q	1.10 (0.70–1.71)	1.31 (0.65–2.64)	1.92 (0.94–3.89)
Estrogen/Progesterone combination: 3q	1.86 (1.26–2.73)***	1.17 (0.52–2.63)	0.81 (0.26–2.54)
Estrogen/Progesterone combination: 4q	1.29 (0.78–2.11)	0.97 (0.36–2.61)	1.24 (0.46–3.35)
Diuretics: 1q	0.82 (0.65–1.03)	0.98 (0.70–1.36)	0.82 (0.47–1.43)
Diuretics: 2q	0.92 (0.74–1.13)	1.43 (1.08–1.88)***	0.98 (0.60–1.61)
Diuretics: 3q	1.23 (1.01–1.51)***	1.67 (1.27–2.20)***	1.94 (1.30–2.89)***
Diuretics: 4q	1.72 (1.42–2.09)^‡^	2.32 (1.76–3.05)***	1.14 (0.65–1.97)
Thiazides: 1q	0.77 (0.56–1.05)	0.88 (0.58–1.33)	1.06 (0.56–2.01)
Thiazides: 2q	1.21 (0.95–1.53)	1.15 (0.81–1.64)	1.03 (0.56–1.90)
Thiazides: 3q	1.68 (1.34–2.11)***	1.36 (0.96–1.93)	2.62 (1.67–4.11)***
Thiazides: 4q	1.43 (1.11–1.83)^‡^	1.31 (0.91–1.90)	1.08 (0.55–2.11)
Loop diuretics: 1q	0.54 (0.37–0.78)^‡^	0.94 (0.61–1.44)	0.43 (0.16–1.16)
Loop diuretics: 2q	0.91 (0.66–1.23)	1.64 (1.16–2.31)***	1.03 (0.50–2.10)
Loop diuretics: 3q	1.01 (0.68–1.48)	1.74 (1.14–2.67)***	0.90 (0.33–2.44)
Loop diuretics: 4q	1.37 (1.05–1.79)^‡^	2.32 (1.68–3.20)^‡^	1.43 (0.78–2.64)
Other diuretics: 1q	0.98 (0.66–1.47)	0.92 (0.53–1.60)	0.95 (0.35–2.58)
Other diuretics: 2q	1.04 (0.71–1.54)	1.68 (1.07–2.64)***	0.48 (0.12–1.92)
Other diuretics: 3q	0.69 (0.42–1.13)	0.82 (0.42–1.59)	1.41 (0.63–3.19)
Other diuretics: 4q	1.36 (0.92–2.01)	1.98 (1.21–3.21)***	0.86 (0.27–2.69)
CCB: 1q	0.82 (0.63–1.06)	0.99 (0.70–1.39)	0.94 (0.52–1.70)
CCB: 2q	0.88 (0.68–1.13)	0.84 (0.58–1.22)	0.95 (0.53–1.72)
CCB: 3q	1.34 (1.06–1.71)***	1.66 (1.20–2.27)***	1.45 (0.84–2.52)
CCB: 4q	1.33 (1.03–1.71)^‡^	1.34 (0.93–1.94)	1.46 (0.83–2.59)
ACEi/ARB: 1q	0.83 (0.65–1.05)	1.10 (0.81–1.50)	0.96 (0.57–1.61)
ACEi/ARB: 2q	0.77 (0.61–0.98)***	0.92 (0.67–1.27)	1.06 (0.65–1.71)
ACEi/ARB: 3q	0.95 (0.76–1.19)	1.08 (0.79–1.48)	1.09 (0.66–1.78)
ACEi/ARB: 4q	1.25 (1.02–1.52)^‡^	1.03 (0.75–1.42)	0.74 (0.41–1.34)
β‐blockers: 1q	0.90 (0.72–1.13)	1.01 (0.73–1.41)	1.24 (0.78–1.98)
β‐blockers: 2q	1.03 (0.84–1.27)	1.04 (0.77–1.41)	0.77 (0.44–1.37)
β‐blockers: 3q	0.81 (0.65–1.02)	0.97 (0.71–1.33)	1.10 (0.68–1.79)
β‐blockers: 4q	1.27 (1.05–1.55)***	1.38 (1.04–1.82)***	1.58 (1.03–2.43)***
PPi: 1q	0.79 (0.64–0.97)***	1.05 (0.78–1.41)	0.71 (0.44–1.16)
PPi: 2q	0.95 (0.78–1.16)	1.09 (0.81–1.46)	1.10 (0.72–1.69)
PPi: 3q	1.12 (0.92–1.37)	1.08 (0.80–1.46)	1.00 (0.63–1.60)
PPi: 4q	1.39 (1.13–1.72)^‡^	1.93 (1.46–2.56)***	1.25 (0.78–2.03)
NSAID: 1q	0.70 (0.57–0.86)^‡^	0.55 (0.39–0.79)^‡^	1.01 (0.68–1.52)
NSAID: 2q	0.96 (0.80–1.15)	1.21 (0.94–1.56)	1.08 (0.73–1.61)
NSAID: 3q	1.25 (1.05–1.48)***	1.05 (0.80–1.37)	1.58 (1.10–2.28)***
NSAID: 4q	1.73 (1.47–2.02)^‡^	1.57 (1.22–2.02)***	1.46 (0.99–2.15)

Abbreviations: β‐blockers, beta‐blockers; ACEi, angiotensin converting enzyme inhibitors; ARB, angiotensin receptor blockers; CCB, calcium channel blockers; NSAID, non‐steroidal anti‐inflammatory drugs; PPi, proton pump inhibitors.

^a^ Group of photosensitizing medications together with daily doses, categorized in quartiles (q).

^b^ Hazard ratio (HR) in relation to never‐use, presented with 95% confidence intervals (CI) regarding basal cell carcinoma (BCC), cutaneous squamous cell carcinoma (cSCC) and cutaneous melanoma (cM).

*** Significant *p* < 0.05. Proportional hazards assumption met.

^‡^ Significant *p* < 0.05. Proportional hazards assumption not met.

Similarly, loop diuretics showed a dose–response trend with the risk of cSCC. Compared to never‐users, the HR gradually increased from 0.94 to 2.3 as the dose increased from low to high. Thiazide diuretics displayed a non‐significant trend of increasing HR of BCC with increasing doses, also seen for cSCC. Moreover, the use of CCB increased the risk of all skin cancers at the two higher dose quartiles, but this was significant only for BCC and, at the second‐highest dosing level, for cSCC. The use of β‐blockers modestly increased the HR of all skin cancers only at the highest dosing levels. The use of ACEi and ARBs was irregularly associated with the risk of BCC, with a significantly decreased risk in the middle‐low quartile dose group and an increased risk in the highest quartile dose group.

Interestingly, low‐dose NSAID significantly decreased the risks of BCC (HR 0.70; 95% CI: 0.57–0.86) and cSCC (HR 0.55; 95% CI: 0.39–0.79), but high‐dose was associated with increased risks of BCC (HR 1.73; 95% CI: 1.47–2.02) and cSCC (HR 1.57; 95% CI: 1.22–2.02). This relationship was not evident when analyzing cM.

## 4 Discussion

In this study, 29,515 prospectively followed women were analyzed regarding pharmaceutical treatments and skin cancer occurrence, while controlling for known risk factors. We found a strong association between estrogen use and the risk of skin cancer, particularly BCC. We also found that the risk of BCC was related to the use of thiazide diuretics, while the risk of cSCC was related to the use of loop diuretics. Furthermore, we found that NSAID use might have a curvilinear association with both BCC and cSCC.

Our major finding highlights the plausible role of female hormones in skin carcinogenetic processes. Estrogen is known to have photosensitizing properties [11, 35], to increase skin thickness, and to improve wound healing in postmenopausal women [36]. Furthermore, several epidemiological studies show a sex‐related difference in incidence and survival after cM. Melanoma incidence is greater in women compared to men before the age of 45 to 65 years, depending on the population, but lower at more advanced ages [37]. Interestingly, the skin and melanocytic lesions have been shown to express estrogen receptors, and cM is the most common cancer during pregnancy [38, 39]. Donley et al. [40] concluded that early menarche and late onset of menopause were associated with a higher risk of cM, implicating that greater estrogen levels over time can infer risk. When separating the hormonal drugs in this study, it became evident that the effect was predominantly due to the estrogen component in hormone treatments. Our results align with the research that implicates estrogen and estrogen/progesterone combination treatments in the risk of developing cM [41, 42, 43], BCC, and cSCC [42, 44, 45]. However, studies are conflicting, and several of those describe no association [40, 46, 47, 48, 49]. We also found that this risk of BCC increased with the dose of estrogen. Dose trends were less convincing for cM and cSCC, which may be due to the low statistical power in these sub‐analyses. A possible limitation of this study is the lack of adjustment for menarche, parity, age at menopause, and use of oral contraceptives. Nevertheless, estrogen use clearly influenced all studied skin cancer types.

The association between diuretics and skin cancer risk has rendered a vast amount of research and scientific discussion [13, 14, 15, 16, 17, 18, 19, 20, 21, 22, 23]. Diuretics is a heterogeneous group of drugs commonly used to treat hypertension, and several have photosensitizing properties [11, 12, 35, 50]. With the stratification of diuretics, we found that loop diuretics were strongly associated with cSCC risk, confirmed by a clear dose–response relationship. These findings do not align with the results of others [13, 14, 17, 21]. Interestingly, loop diuretics did not affect the risks of BCC or cM, contradictory to one study, which showed an increased risk for cM [20]. Emerging evidence has resulted in a move by the FDA to change the labelling of HCTZ [24], highlighting a slightly increased risk of non‐melanoma skin cancer; no recommendation was made to stop its use. Several studies have shown an association between thiazides, specifically HCTZ, and the risk of skin cancer [16, 18, 19, 22, 51, 52]. We also found a correlation between thiazide use and BCC (HR 1.25; 95% CI: 1.09–1.44) with evidence of a dose–response relationship. We further found a similar result for cM (HR 1.41 for ever use), but no dose–response relationship. This agrees with the meta‐analysis by Gandini et al. [14] Surprisingly, and in contrast to previous meta‐analyses [16, 22, 25], we found no significant association between thiazide use and the risk of cSCC, which is commonly associated with thiazide use. Unfortunately, our results do not settle the debate about thiazide use and the risk of skin cancer. It is important to consider the overall health benefits of thiazide use before recommending any changes to an antihypertensive treatment, given that the overall benefits likely outweigh the small risks of skin cancer.

Varying results have also been found for two other large aggregates of antihypertensive drugs: β‐blockers and CCB [17, 21, 43, 53, 54]. Two meta‐analyses have been published to date. Gandini [14] showed an increased risk of all three skin cancers with the intake of CCB, and Tang [23] showed an increased risk of cM with β‐blocker use. Our results for CCB and β‐blockers were not convincing, probably due to a lack of statistical power in the analyses of CCB and the risk of cSCC and cM. If anything, the results point to risk being associated with increased CCB and β‐blocker doses. However, these results could be confounded by polypharmacy, and the risks associated with other pharmaceuticals, such as diuretics, could increase with combination treatments in these groups.

Both ARB and ACEi have photosensitizing abilities [9, 10, 11, 12]. Schmidt et al. [21] without adjustment for lifestyle factors or phenotypic traits, found an increased risk of cM with long‐term use of ARB, but not with ACEi. To date, little evidence of an association between ACEi/ARB risk of skin cancer is available. Instead, a protective effect has been suggested due to inhibition of tumour growth and angiogenesis [55, 56, 57]. We found neither an increase nor a decrease in skin cancer risk with the use of ACEi/ARB. Grouping these drugs did not allow us to draw conclusions about them separately. Nonetheless, our findings confirm the results of many others [14, 21, 23, 52, 53, 54, 56, 57, 58, 59, 60].

Few studies have attempted to find an association between antibiotic use and the risk of skin cancer. Although they are photosensitizing, antibiotics are often used for short periods, and analyses are challenging. Our analyses revealed a positive association between ever use of these antibiotics and the risk of BCC and cSCC, but not cM. Due to the heterogeneity of this group of drugs, where various dosing schedules are employed, no dose–response analysis was performed. Our results agree with those of Kaae et al. who studied the effect of short‐term drug use [17]. Other studies have shown that tetracyclines are associated with BCC, but not cSCC or cM [61], and that quinolones are associated with cM risk [62]. It is difficult to draw firm conclusions from our results, and this relationship needs further investigation with careful sub‐analyses of the constituents in this group.

We found that the common antidiabetic drugs metformin and glibenclamide were unrelated to skin cancer risk, which contrasts with Chang and collegues [63], who found a modestly reduced risk of skin cancer with metformin use. Use of PPi, which also has photosensitizing properties [64], particularly drew our interest since the literature provides very little insight. We found that the HR for the risk of cSCC was increased with PPi use; however, adjusting for multiple testing, the significance was lost, and no dose–response relationship was found. Again, our results could be confounded by polypharmacy, and further research is warranted.

Results of studies on the relationship between NSAID use and risk of skin cancer have shown divergent results [62, 65, 66, 67]. Our ever‐never analysis showed no association between skin cancer and NSAID use, except possibly in a curvilinear way, particularly for BCC. In a meta‐analysis performed by Ma et al. [67] NSAIDs were found to be significantly associated with BCC and SCC, but not with cM. Notably, we found an increased risk of skin cancer in the higher dose groups, but this result could be confounded by unregistered associated autoimmune processes such as arthritis.

## 5 Strengths and Limitations

A substantial strength of this study is that our cohort was followed prospectively with meticulous collection of unbiased information on known risk factors for skin cancer such as phenotypic traits and UVR exposure. Additionally, validated questionnaires and population‐based registries were used to assess UVR exposure and outcomes. Consequently, our data is population‐based, valid, and generalizable to other women in comparable settings.

Comparable research in the field is primarily retrospective, imparting a risk for recall bias, often without adjustments for risk factors that are immensely important when investigating photosensitizing drugs that exert their effects by interacting with UVR. Moreover, other studies often include methodological limitations such as grouping different types of skin cancer or different drugs [15, 56, 59, 60], short follow‐up times [15, 57] and suboptimal control of confounders [13, 17, 19, 21, 23, 50, 58] such as sun sensitivity and sun exposure. Indeed, pharmacological studies are complex and prone to confounding factors and bias, and ongoing discussions focus on statistical methods, interpretation, and publication bias [25].

This study also has limitations. First, the generalization of its results is hampered by including women from only a single geographic area. Second, our information about drug intake is limited to the drugs that were prescribed and dispensed; we cannot be certain the participants in the study consumed the drugs as prescribed. Third, several drugs were grouped and not assessed separately; therefore, solid conclusions could not be made for the individual drugs. Fourth, we did not consider the confounding effects of associated treatments, which are relevant in an aging population where both polypharmacy and skin cancer are common. Also, confounding by indication is possible, given that individuals with underlying diseases are known to be at a greater risk of skin cancer diagnosis, given their increased frequency of visits to their physicians. Fifth, we recognize that hazard ratios between examined drugs and skin cancer risk are modest and must be considered in relation to health benefits from the treatment of the prescribed drugs. Lastly, we used exposure information from the baseline questionnaire, but did not consider possible behavioral changes that could affect the outcome.

## 6 Conclusion

We found that estrogen use increases the risk of skin cancer. The use of loop diuretics increases the risk of cSCC, and the use of thiazides increases the risk of BCC. We suggest that physicians advise their female patients who are treated with estrogen, thiazides, or loop diuretics to limit their sun exposure. We do not recommend discontinuation of any of the medications studied in this paper. Future well‐designed prospective studies are warranted to further investigate the relationships between common photosensitizing drugs and the risk of skin cancer.

## Author Contributions

Gustav Boelsgaard Christensen, Kari Nielsen, Jenny Sandgren, and Åsa Ingvar had full access to all of the study's data and are responsible for its integrity and the accuracy of the data analysis. Concept and design: Gustav Boelsgaard Christensen, Kari Nielsen, and Åsa Ingvar. Acquisition, analysis, or interpretation of data: All authors. Drafting of the manuscript: Gustav Boelsgaard Christensen. Critical review of the manuscript for important intellectual content: All authors. Statistical analysis: Jenny Sandgren, Åsa Ingvar and Kari Nielsen. Obtained funding: Kari Nielsen and Åsa Ingvar. Administrative, technical, or material support: Kari Nielsen and Åsa Ingvar. Supervision: Kari Nielsen and Åsa Ingvar.

## Ethics Statement

The Institutional Ethics Review Board of Lund University and the Swedish Ethical Review Authority approved the study (LU‐34‐1992, LU‐632‐2003, LU‐781‐2005, LU‐849‐2005, LU‐190‐2007, LU‐644‐2015, and LU‐02914‐2022).

## Conflicts of Interest

G.B.C. has received speaker honoraria from LEO Pharma and UCB Pharma. K.N. has received speaker honoraria from Galderma Sweden, LEO Pharma, Novartis Sweden, and UCB Pharma and has served on one advisory board for MSD. Å.I. has received speaker and consulting honoraria from Galderma Sweden, Perrigo Sweden, MSD Sweden, and Biofrontera Sweden. J.K. has received speaker honoraria from Galderma Sweden and Sanofi Sweden. The other authors declare no conflicts of interest. The companies providing honoraria did not influence the design, data collection, analysis, interpretation, or reporting of the study.

## Supporting information


**Table S1.** Number of persons diagnosed with respective skin cancer type, and risk of skin cancer development (assessed with multivariate Cox‐regression models), categorized by phenotypic and environmental factors.

## Data Availability

The data underlying this article will be shared on reasonable request to the corresponding author. Necessary approvals for data transfers must also be obtained.

## References

[1] A. E. Cust , B. K. Armstrong , C. Goumas , et al., “Sunbed Use During Adolescence and Early Adulthood Is Associated With Increased Risk of Early‐Onset Melanoma,” International Journal of Cancer 128 (2011): 2425–2435. 20669232 10.1002/ijc.25576 PMC2993823

[2] G. B. Christensen , C. Ingvar , L. W. Hartman , H. Olsson , and K. Nielsen , “Sunbed Use Increases Cutaneous Squamous Cell Carcinoma Risk in Women: A Large‐Scale, Prospective Study in Sweden,” Acta Dermato‐Venereologica 99 (2019): 878–883. 31017252 10.2340/00015555-3198

[3] I. Stanganelli , S. Gandini , S. Magi , et al., “Sunbed Use Among Subjects at High Risk of Melanoma: An Italian Survey After the Ban,” British Journal of Dermatology 169 (2013): 351–357. 23601037 10.1111/bjd.12384

[4] B. K. Armstrong and A. Kricker , “The Epidemiology of UV Induced Skin Cancer,” Journal of Photochemistry and Photobiology. B, Biology 63 (2001): 8–18. 11684447 10.1016/s1011-1344(01)00198-1

[5] J. F. Thompson , R. A. Scolyer , and R. F. Kefford , “Cutaneous Melanoma,” Lancet 365 (2005): 687–701. 15721476 10.1016/S0140-6736(05)17951-3

[6] V. Madan , J. T. Lear , and R. M. Szeimies , “Non‐Melanoma Skin Cancer,” Lancet 375 (2010): 673–685. 20171403 10.1016/S0140-6736(09)61196-X

[7] F. Erdmann , J. Lortet‐Tieulent , J. Schuz , et al., “International Trends in the Incidence of Malignant Melanoma 1953–2008—Are Recent Generations at Higher or Lower Risk?,” International Journal of Cancer 132 (2013): 385–400. 22532371 10.1002/ijc.27616

[8] A. Lomas , J. Leonardi‐Bee , and F. Bath‐Hextall , “A Systematic Review of Worldwide Incidence of Nonmelanoma Skin Cancer,” British Journal of Dermatology 166 (2012): 1069–1080. 22251204 10.1111/j.1365-2133.2012.10830.x

[9] L. Lankerani and E. D. Baron , “Photosensitivity to Exogenous Agents,” Journal of Cutaneous Medicine and Surgery 8 (2004): 424–431. 15988550 10.1007/s10227-005-0017-3

[10] A. F. Monteiro , M. Rato , and C. Martins , “Drug‐Induced Photosensitivity: Photoallergic and Phototoxic Reactions,” Clinics in Dermatology 34 (2016): 571–581. 27638435 10.1016/j.clindermatol.2016.05.006

[11] D. E. Moore , “Drug‐Induced Cutaneous Photosensitivity: Incidence, Mechanism, Prevention and Management,” Drug Safety 25 (2002): 345–372. 12020173 10.2165/00002018-200225050-00004

[12] F. Lozzi , C. Di Raimondo , C. Lanna , et al., “Latest Evidence Regarding the Effects of Photosensitive Drugs on the Skin: Pathogenetic Mechanisms and Clinical Manifestations,” Pharmaceutics 12 (2020): 12. 10.3390/pharmaceutics12111104 PMC7698592 33213076

[13] A. O. Jensen , H. F. Thomsen , M. C. Engebjerg , A. B. Olesen , H. T. Sørensen , and M. R. Karagas , “Use of Photosensitising Diuretics and Risk of Skin Cancer: A Population‐Based Case‐Control Study,” British Journal of Cancer 99 (2008): 1522–1528. 18813314 10.1038/sj.bjc.6604686 PMC2579687

[14] S. Gandini , D. Palli , G. Spadola , et al., “Anti‐Hypertensive Drugs and Skin Cancer Risk: A Review of the Literature and Meta‐Analysis,” Critical Reviews in Oncology/Hematology 122 (2018): 1–9. 29458778 10.1016/j.critrevonc.2017.12.003

[15] E. McDonald , D. M. Freedman , B. H. Alexander , et al., “Prescription Diuretic Use and Risk of Basal Cell Carcinoma in the Nationwide U.S. Radiologic Technologists Cohort,” Cancer Epidemiology, Biomarkers & Prevention 23 (2014): 1539–1545. 24812037 10.1158/1055-9965.EPI-14-0251 PMC4119543

[16] S. Nochaiwong , M. Chuamanochan , C. Ruengorn , et al., “Use of Thiazide Diuretics and Risk of all Types of Skin Cancers: An Updated Systematic Review and Meta‐Analysis,” Cancers 14, no. 10 (2022): 2566, 10.3390/cancers14102566. 35626169 PMC9140049

[17] J. Kaae , H. A. Boyd , A. V. Hansen , H. C. Wulf , J. Wohlfahrt , and M. Melbye , “Photosensitizing Medication Use and Risk of Skin Cancer,” Cancer Epidemiology, Biomarkers & Prevention 19 (2010): 2942–2949. 20861398 10.1158/1055-9965.EPI-10-0652

[18] S. A. Pedersen , D. Gaist , S. A. J. Schmidt , L. R. Hölmich , S. Friis , and A. Pottegård , “Hydrochlorothiazide Use and Risk of Nonmelanoma Skin Cancer: A Nationwide Case‐Control Study From Denmark,” Journal of the American Academy of Dermatology 78 (2018): 673.e9–681.e9. 29217346 10.1016/j.jaad.2017.11.042

[19] A. Pottegard , S. A. Pedersen , S. A. J. Schmidt , et al., “Association of Hydrochlorothiazide Use and Risk of Malignant Melanoma,” JAMA Internal Medicine 178 (2018): 1120–1122. 29813157 10.1001/jamainternmed.2018.1652 PMC6143099

[20] R. Ruiter , L. E. Visser , M. Eijgelsheim , et al., “High‐Ceiling Diuretics Are Associated With an Increased Risk of Basal Cell Carcinoma in a Population‐Based Follow‐Up Study,” European Journal of Cancer 46 (2010): 2467–2472. 20605443 10.1016/j.ejca.2010.04.024

[21] S. A. Schmidt , M. Schmidt , F. Mehnert , S. Lemeshow , and H. T. Sørensen , “Use of Antihypertensive Drugs and Risk of Skin Cancer,” Journal of the European Academy of Dermatology and Venereology 29, no. 8 (2015): 1545–1554, 10.1111/jdv.12921. 25640031

[22] S. C. Shao , C. C. Lai , Y. H. Chen , E. C. C. Lai , M. J. Hung , and C. C. Chi , “Associations of Thiazide Use With Skin Cancers: A Systematic Review and Meta‐Analysis,” BMC Medicine 20 (2022): 228. 35794547 10.1186/s12916-022-02419-9 PMC9260996

[23] H. Tang , S. Fu , S. Zhai , Y. Song , and J. Han , “Use of Antihypertensive Drugs and Risk of Malignant Melanoma: A Meta‐Analysis of Observational Studies,” Drug Safety 41 (2018): 161–169. 28905299 10.1007/s40264-017-0599-x

[24] U.S. Food and Drug Administration , “FDA Approves Label Changes to Hydrochlorothiazide to Describe Small Risk of Non‐Melanoma Skin Cancer,” 2022.

[25] B. Bendinelli , G. Masala , G. Garamella , D. Palli , and S. Caini , “Do Thiazide Diuretics Increase the Risk of Skin Cancer? A Critical Review of the Scientific Evidence and Updated Meta‐Analysis,” Current Cardiology Reports 21 (2019): 92. 31352643 10.1007/s11886-019-1183-z

[26] E. Weidman‐Evans , A. Rhodes , and L. Ferrington , “What Is the Relationship Between Photosensitizing Drugs and Skin Cancer?,” JAAPA 36 (2023): 8–10. 10.1097/01.JAA.0000931448.63999.de 37229580

[27] A. M. Ingvar , H. Olsson , P. Broberg , et al., “Participation in a Prospective Cohort Study on Melanoma Did Not Affect the Incidence and Mortality of the Studied Disease,” Acta Dermato‐Venereologica 100 (2020): adv00010. 31663602 10.2340/00015555-3362 PMC9128912

[28] P. G. Lindqvist , E. Epstein , M. Landin‐Olsson , M. Åkerlund , and H. Olsson , “Women With Fair Phenotypes Seem to Confer a Survival Advantage in a Low UV Milieu. A Nested Matched Case Control Study,” PLoS One 15 (2020): e0228582. 31999788 10.1371/journal.pone.0228582 PMC6992199

[29] P. G. Lindqvist , E. Epstein , M. Landin‐Olsson , et al., “Avoidance of Sun Exposure Is a Risk Factor for All‐Cause Mortality: Results From the Melanoma in Southern Sweden Cohort,” Journal of Internal Medicine 276 (2014): 77–86. 24697969 10.1111/joim.12251

[30] K. Nielsen , A. Masback , H. Olsson , et al., “A Prospective, Population‐Based Study of 40,000 Women Regarding Host Factors, UV Exposure and Sunbed Use in Relation to Risk and Anatomic Site of Cutaneous Melanoma,” International Journal of Cancer 131 (2012): 706–715. 21898390 10.1002/ijc.26408

[31] A. Masback , J. Westerdahl , C. Ingvar , et al., “Clinical and Histopathological Characteristics in Relation to Aetiological Risk Factors in Cutaneous Melanoma: A Population‐Based Study,” Melanoma Research 9 (1999): 189–197. 10380942 10.1097/00008390-199904000-00012

[32] P. G. Lindqvist , E. Epstein , K. Nielsen , M. Landin‐Olsson , C. Ingvar , and H. Olsson , “Avoidance of Sun Exposure as a Risk Factor for Major Causes of Death: A Competing Risk Analysis of the Melanoma in Southern Sweden Cohort,” Journal of Internal Medicine 280 (2016): 375–387. 26992108 10.1111/joim.12496

[33] J. Westerdahl , H. Anderson , H. Olsson , et al., “Reproducibility of a Self‐Administered Questionnaire for Assessment of Melanoma Risk,” International Journal of Epidemiology 25 (1996): 245–251. 9119548 10.1093/ije/25.2.245

[34] SAS Institute Inc , SAS Enterprise Guide 8.3: User's Guide (SAS Institute Inc., 2020).

[35] R. S. Stern , “Photocarcinogenicity of Drugs,” Toxicology Letters 102–103 (1998): 389–392. 10.1016/s0378-4274(98)00237-9 10022284

[36] M. P. Brincat , Y. M. Baron , and R. Galea , “Estrogens and the Skin,” Climacteric 8 (2005): 110–123. 16096167 10.1080/13697130500118100

[37] C. M. Olsen , J. F. Thompson , N. Pandeya , and D. C. Whiteman , “Evaluation of Sex‐Specific Incidence of Melanoma,” JAMA Dermatology 156 (2020): 553–560. 32211827 10.1001/jamadermatol.2020.0470 PMC7097866

[38] T. M. Andersson , A. L. Johansson , I. Fredriksson , and M. Lambe , “Cancer During Pregnancy and the Postpartum Period: A Population‐Based Study,” Cancer 121, no. 12 (2015): 2072–2077, 10.1002/cncr.29325. 25737403

[39] D. L. Ellis , R. G. Wheeland , and H. Solomon , “Estrogen and Progesterone Receptors in Primary Cutaneous Melanoma,” Journal of Dermatologic Surgery and Oncology 11 (1985): 54–59. 3965520 10.1111/j.1524-4725.1985.tb02891.x

[40] G. M. Donley , W. T. Liu , R. M. Pfeiffer , et al., “Reproductive Factors, Exogenous Hormone Use and Incidence of Melanoma Among Women in the United States,” British Journal of Cancer 120 (2019): 754–760. 30814688 10.1038/s41416-019-0411-z PMC6461881

[41] E. R. Koomen , A. Joosse , R. M. Herings , et al., “Estrogens, Oral Contraceptives and Hormonal Replacement Therapy Increase the Incidence of Cutaneous Melanoma: A Population‐Based Case‐Control Study,” Annals of Oncology 20 (2009): 358–364. 18725391 10.1093/annonc/mdn589

[42] K. Lallas , P. Anagnostis , P. Theocharis , et al., “The Effect of Menopausal Hormone Therapy on the Risk of Melanoma and Keratinocyte Skin Cancer: A Systematic Review and Meta‐Analysis of Observational Studies,” Maturitas 168 (2023): 20–28. 36372010 10.1016/j.maturitas.2022.10.010

[43] J. Westerdahl , H. Olsson , A. Masback , et al., “Risk of Malignant Melanoma in Relation to Drug Intake, Alcohol, Smoking and Hormonal Factors,” British Journal of Cancer 73 (1996): 1126–1131. 8624275 10.1038/bjc.1996.216 PMC2074414

[44] E. K. Cahoon , C. M. Kitahara , E. Ntowe , et al., “Female Estrogen‐Related Factors and Incidence of Basal Cell Carcinoma in a Nationwide US Cohort,” Journal of Clinical Oncology 33 (2015): 4058–4065. 26527779 10.1200/JCO.2015.62.0625 PMC4669591

[45] F. Birch‐Johansen , A. Jensen , A. B. Olesen , J. Christensen , A. Tjønneland , and S. K. Kjær , “Does Hormone Replacement Therapy and Use of Oral Contraceptives Increase the Risk of Non‐Melanoma Skin Cancer?,” Cancer Causes & Control 23 (2012): 379–388. 22215431 10.1007/s10552-011-9887-4

[46] J. Y. Tang , K. M. Spaunhurst , R. T. Chlebowski , et al., “Menopausal Hormone Therapy and Risks of Melanoma and Nonmelanoma Skin Cancers: Women's Health Initiative Randomized Trials,” Journal of the National Cancer Institute 103 (2011): 1469–1475. 21878677 10.1093/jnci/djr333 PMC3186783

[47] C. M. Olsen , N. Pandeya , B. S. Thompson , et al., “Hormonal and Reproductive Factors and Incidence of Basal Cell Carcinoma and Squamous Cell Carcinoma in a Large, Prospective Cohort,” Journal of the American Academy of Dermatology 78 (2018): 615.e2–618.e2. 28947292 10.1016/j.jaad.2017.09.033

[48] C. M. Olsen , N. Pandeya , J. C. Dusingize , et al., “Reproductive Factors, Hormone Use and Melanoma Risk: An Australian Prospective Cohort Study,” British Journal of Dermatology 184 (2021): 361–363. 32856295 10.1111/bjd.19498

[49] S. Gandini , S. Iodice , E. Koomen , A. D. Pietro , F. Sera , and S. Caini , “Hormonal and Reproductive Factors in Relation to Melanoma in Women: Current Review and Meta‐Analysis,” European Journal of Cancer 47 (2011): 2607–2617. 21620689 10.1016/j.ejca.2011.04.023

[50] R. Kreutz , E. A. H. Algharably , and A. Douros , “Reviewing the Effects of Thiazide and Thiazide‐Like Diuretics as Photosensitizing Drugs on the Risk of Skin Cancer,” Journal of Hypertension 37 (2019): 1950–1958. 31145177 10.1097/HJH.0000000000002136

[51] E. Eworuke , N. Haug , M. Bradley , et al., “Risk of Nonmelanoma Skin Cancer in Association With Use of Hydrochlorothiazide‐Containing Products in the United States,” JNCI Cancer Spectrum 5 (2021): pkab009. 33733052 10.1093/jncics/pkab009 PMC7947823

[52] A. M. Drucker , L. Hollestein , Y. Na , et al., “Association Between Antihypertensive Medications and Risk of Skin Cancer in People Older Than 65 Years: A Population‐Based Study,” CMAJ 193, no. 15 (2021): E508–E516, 10.1503/cmaj.201971. 33846199 PMC8087333

[53] L. H. Lindholm , H. Anderson , T. Ekbom , et al., “Relation Between Drug Treatment and Cancer in Hypertensives in the Swedish Trial in Old Patients With Hypertension 2: A 5‐Year, Prospective, Randomised, Controlled Trial,” Lancet 358 (2001): 539–544. 11520524 10.1016/s0140-6736(01)05704-x

[54] L. Rosenberg , R. S. Rao , J. R. Palmer , et al., “Calcium Channel Blockers and the Risk of Cancer,” JAMA 279, no. 13 (1998): 1000–1004, 10.1001/jama.279.13.1000. 9533498

[55] T. Rosenthal and I. Gavras , “Angiotensin Inhibition and Malignancies: A Review,” Journal of Human Hypertension 23 (2009): 623–635. 19339998 10.1038/jhh.2009.21

[56] A. F. Lever , D. J. Hole , C. R. Gillis , et al., “Do Inhibitors of Angiotensin‐I‐Converting Enzyme Protect Against Risk of Cancer?,” Lancet 352 (1998): 179–184. 9683206 10.1016/S0140-6736(98)03228-0

[57] E. R. Koomen , R. M. Herings , H. J. Guchelaar , and T. Nijsten , “Melanoma Incidence and Exposure to Angiotensin‐Converting Enzyme Inhibitors and Angiotensin Receptor Blockers,” Cancer Epidemiology 33, no. 5 (2009): 391–395, 10.1016/j.canep.2009.10.005. 19896919

[58] B. Nardone , S. Majewski , A. S. Kim , et al., “Melanoma and Non‐Melanoma Skin Cancer Associated With Angiotensin‐Converting‐Enzyme Inhibitors, Angiotensin‐Receptor Blockers and Thiazides: A Matched Cohort Study,” Drug Safety 40 (2017): 249–255. 27943160 10.1007/s40264-016-0487-9

[59] S. Friis , H. T. Sorensen , L. Mellemkjaer , et al., “Angiotensin‐Converting Enzyme Inhibitors and the Risk of Cancer: A Population‐Based Cohort Study in Denmark,” Cancer 92 (2001): 2462–2470. 11745304 10.1002/1097-0142(20011101)92:9<2462::aid-cncr1596>3.0.co;2-l

[60] J. B. Christian , K. L. Lapane , A. L. Hume , C. B. Eaton , M. A. Weinstock , and VATTC Trial , “Association of ACE Inhibitors and Angiotensin Receptor Blockers With Keratinocyte Cancer Prevention in the Randomized VATTC Trial,” Journal of the National Cancer Institute 100 (2008): 1223–1232. 18728281 10.1093/jnci/djn262

[61] W. Q. Li , A. M. Drucker , E. Cho , et al., “Tetracycline Use and Risk of Incident Skin Cancer: A Prospective Study,” British Journal of Cancer 118 (2018): 294–298. 29073637 10.1038/bjc.2017.378 PMC5785738

[62] S. J. Siiskonen , E. R. Koomen , L. E. Visser , et al., “Exposure to Phototoxic NSAIDs and Quinolones Is Associated With an Increased Risk of Melanoma,” European Journal of Clinical Pharmacology 69 (2013): 1437–1444. 23471440 10.1007/s00228-013-1476-x

[63] M. S. Chang , R. I. Hartman , J. Xue , E. L. Giovannucci , H. Nan , and K. Yang , “Risk of Skin Cancer Associated With Metformin Use: A Meta‐Analysis of Randomized Controlled Trials and Observational Studies,” Cancer Prevention Research 14 (2021): 77–84. 32958585 10.1158/1940-6207.CAPR-20-0376

[64] A. Alrashidi , L. E. Rhodes , J. C. H. Sharif , F. C. Kreeshan , M. D. Farrar , and T. Ahad , “Systemic Drug Photosensitivity‐Culprits, Impact and Investigation in 122 Patients,” Photodermatology, Photoimmunology & Photomedicine 36 (2020): 441–451. 32564400 10.1111/phpp.12583

[65] E. A. George , N. Baranwal , J. H. Kang , A. A. Qureshi , A. M. Drucker , and E. Cho , “Photosensitizing Medications and Skin Cancer: A Comprehensive Review,” Cancers 13, no. 10 (2021): 2344, 10.3390/cancers13102344. 34066301 PMC8152064

[66] A. Joosse , E. R. Koomen , M. K. Casparie , R. M. C. Herings , H. J. Guchelaar , and T. Nijsten , “Non‐Steroidal Anti‐Inflammatory Drugs and Melanoma Risk: Large Dutch Population‐Based Case‐Control Study,” Journal of Investigative Dermatology 129 (2009): 2620–2627. 19587697 10.1038/jid.2009.201

[67] Y. Ma , P. Yu , S. Lin , Q. Li , Z. Fang , and Z. Huang , “The Association Between Nonsteroidal Anti‐Inflammatory Drugs and Skin Cancer: Different Responses in American and European Populations,” Pharmacological Research 152 (2020): 104499. 31689521 10.1016/j.phrs.2019.104499

