# Supplementary material for: Photosensitizing Drugs and Risk of Skin Cancer in Women—A Prospective Population‐Based Study
Source: Photodermatol Photoimmunol Photomed. 2025 Mar 18;41(2):e70013. doi: 10.1111/phpp.70013 (PMC11919808; doi:10.1111/phpp.70013)
Supplement: Supplementary file 1 — Table S1. Number of persons diagnosed with respective skin cancer type, and risk of skin cancer development (assessed with multivariate Cox‐regression models), categorized by phenotypic and environmental factors. [file PHPP-41-e70013-s001.docx]

| **Table S1.** Number of persons diagnosed with respective skin cancer type, and risk of skin cancer development (assessed with multivariate Cox-regression models), categorized by phenotypic and environmental factors. | | | | | | | | |
| --- | --- | --- | --- | --- | --- | --- | --- | --- |
| **Variables^+^** | **Overall, n (%^*^)** | **Persons without skin cancer, n^**^ (%^*^)** | **BCC, n** | **HR (95% CI) ^§^** | **cSCC, n** | **HR (95% CI) ^§^** | **cM, n** | **HR (95% CI) ^§^** |
| *Age* |  |  |  |  |  |  |  |  |
| 40–49 | 4493 (21.3) | 4298 (22.4) | 127 | ref | 34 | ref | 43 | ref |
| 50–59 | 5961 (28.3) | 5594 (29.2) | 267 | 1.61 (1.30 – 1.99) | 64 | 1.43 (0.94 – 2.17) | 69 | 1.22 (0.83 – 1.79) |
| 60–69 | 5421 (25.7) | 4801 (25.0) | 460 | 3.20 (2.63 – 3.89) | 158 | 4.03 (2.78 – 5.84) | 78 | 1.56 (1.08 – 2.26) |
| 70–79 | 4274 (20.3) | 3700 (19.3) | 381 | 3.70 (3.02 – 4.52) | 209 | 7.61 (5.30 – 10.94) | 61 | 1.71 (1.16 – 2.53) |
| >80 | 913 (4.3) | 794 (4.1) | 73 | 3.99 (2.99 – 5.32) | 63 | 13.53 (8.91 – 20.55) | 6 | 0.96 (0.41 – 2.25) |
| *Nevi left arm* |  |  |  |  |  |  |  |  |
| ≤ 10 | 19945 (94.7) | 18143 (94.6) | 1254 | Ref | 518 | ref | 244 | ref |
| > 10 | 1117 (5.3) | 1044 (5.4) | 54 | 0.73 (0.56 – 0.96) | 10 | 0.33 (0.17 – 0.61) | 13 | 0.91 (0.52 – 1.59) |
| *Freckles* |  |  |  |  |  |  |  |  |
| No | 11911 (56.6) | 10915 (56.9) | 704 | ref | 262 | ref | 130 | ref |
| Yes | 9151 (43.4) | 8272 (43.1) | 604 | 1.10 (0.99 – 1.23) | 266 | 1.30 (1.10 – 1.54) | 127 | 1.25 (0.98 – 1.60) |
| *Hair color* |  |  |  |  |  |  |  |  |
| Black, brown | 5075 (24.1) | 4706 (24.5) | 267 | ref | 89 | ref | 50 | ref |
| Dark/medium blonde, grizzled, gray | 13706 (65.1) | 12450 (64.9) | 873 | 1.24 (1.08 – 1.42) | 365 | 1.55 (1.23 – 1.96) | 165 | 1.25 (0.91 – 1.71) |
| Red, light blonde, white | 2281 (10.8) | 2031 (10.6) | 168 | 1.42 (1.17 – 1.73) | 74 | 1.88 (1.38 – 2.56) | 42 | 1.90 (1.26 – 2.86) |
| *Light eye color* |  |  |  |  |  |  |  |  |
| No | 10093 (47.9) | 9221 (48.1) | 605 | ref | 237 | ref | 121 | ref |
| Yes | 10969 (52.1) | 9966 (51.9) | 703 | 1.08 (0.97 – 1.20) | 291 | 1.14 (0.96 – 1.35) | 136 | 1.04 (0.82 – 1.33) |
| *First-degree relative with cM* |  |  |  |  |  |  |  |  |
| No | 19824 (94.1) | 18098 (94.3) | 1196 | ref | 489 | ref | 238 | ref |
| Yes | 1238 (5.9) | 1089 (5.7) | 112 | 1.53 (1.26 – 1.85) | 39 | 1.28 (0.93 – 1.78) | 19 | 1.29 (0.81 – 2.05) |
| *Smoking* |  |  |  |  |  |  |  |  |
| Never | 8284 (39.3) | 7479 (39.0) | 566 | ref | 235 | ref | 114 | ref |
| Ever | 12778 (60.7) | 11708 (61.0) | 742 | 0.85 (0.76 – 0.95) | 293 | 0.81 (0.68 – 0.96) | 143 | 0.82 (0.64 – 1.04) |
| *Burn when sunbathing* |  |  |  |  |  |  |  |  |
| Never | 10209 (48.5) | 9292 (48.4) | 645 | ref | 282 | ref | 103 | ref |
| 1-2 times/year | 10106 (48.0) | 9223 (48.1) | 612 | 0.91 (0.81 – 1.02) | 222 | 0.75 (0.63 – 0.90) | 140 | 1.30 (1.01 – 1.68) |
| > 3 times/year | 747 (3.5) | 672 (3.5) | 51 | 1.03 (0.77 – 1.37) | 24 | 1.10 (0.72 – 1.67) | 14 | 1.77 (1.01 – 3.09) |
| *Sunbathing during summer* |  |  |  |  |  |  |  |  |
| Never | 741 (3.5) | 699 (3.6) | 27 | ref | 17 | ref | 4 | ref |
| 1-14 times/year | 7525 (35.7) | 6907 (36.0) | 429 | 1.42 (0.96 – 2.09) | 174 | 0.89 (0.54 – 1.46) | 83 | 1.82 (0.67 – 4.96) |
| > 15 times/year | 12796 (60.8) | 11581 (60.4) | 852 | 1.65 (1.12 – 2.41) | 337 | 1.00 (0.61 – 1.63) | 170 | 2.17 (0.80 – 5.84) |
| *Go abroad on sunny vacations* |  |  |  |  |  |  |  |  |
| Never | 8587 (40.8) | 7926 (41.3) | 454 | ref | 203 | ref | 77 | ref |
| 1-10 days/year | 12081 (57.4) | 10903 (56.8) | 831 | 1.29 (1.15 – 1.45) | 311 | 1.07 (0.90 – 1.28) | 176 | 1.60 (1.22 – 2.09) |
| > 10 days/year | 394 (1.9) | 358 (1.9) | 23 | 1.11 (0.73 – 1.68) | 14 | 1.52 (0.89 – 2.62) | 4 | 1.14 (0.42 – 3.11) |
| *Higher education* |  |  |  |  |  |  |  |  |
| No | 14196 (67.4) | 12954 (67.5) | 857 | ref | 364 | ref | 166 | ref |
| Yes | 6866 (32.6) | 6233 (32.5) | 451 | 1.06 (0.94 – 1.19) | 164 | 0.90 (0.75 – 1.08) | 91 | 1.10 (0.85 – 1.42) |
| *Working outdoors* |  |  |  |  |  |  |  |  |
| No | 15437 (73.3) | 14007 (73.0) | 999 | ref | 399 | ref | 194 | ref |
| Yes | 5625 (26.7) | 5180 (27.0) | 309 | 0.84 (0.74 – 0.96) | 129 | 0.88 (0.73 – 1.08) | 63 | 0.89 (0.67 – 1.18) |
| *Use of sunbeds/UV-lamps* |  |  |  |  |  |  |  |  |
| No | 10468 (49.7) | 9515 (49.6) | 669 | ref | 281 | ref | 106 | ref |
| Yes | 10594 (50.3) | 9672 (50.4) | 639 | 0.91 (0.81 – 1.01) | 247 | 0.83 (0.70 – 0.99) | 151 | 1.36 (1.06 – 1.74) |
| **^+^**Variables according to questions in the questionnaire.  ^*^Proportion of individuals within the category group.  ^**^Individuals in the cohort, not diagnosed with a skin cancer.  **^§^** Hazard ratio (HR) in relation to reference group, presented with a 95% confidence interval (CI) regarding basal cell carcinoma (BCC), cutaneous squamous cell carcinoma (cSCC) and cutaneous melanoma (cM).  UV (ultra violet). | | | | | | | | |
